# Beliefs and perceptions of genetic testing by clinical, demographic, and health characteristics: A U.S.-based study utilizing the health information national trends (HINTS) survey

**DOI:** 10.1016/j.pmedr.2025.103204

**Published:** 2025-08-08

**Authors:** Pranali G. Patel, Marvin E. Langston, Justin X. Moore, Yunan Han, Marquita W. Lewis-Thames, Lindsay Fuzzell, Saira Khan

**Affiliations:** a Department of Epidemiology, The University of Alabama at Birmingham, Birmingham, AL, USA; b Department of Epidemiology, Saint Louis University, St. Louis, MO, USA; c Department of Epidemiology and Population Health, Stanford University School of Medicine, Palo Alto, California, USA; d Center for Health Equity Transformation, Department of Behavioral Science, College of Medicine, University of Kentucky, Lexington, KY, USA; e Department of Internal Medicine, College of Medicine, University of Kentucky, Lexington, KY, USA; f Division of General Surgery, The Brooklyn Hospital Center, Brooklyn, New York, USA; g Department of Medical Social Science, Feinberg School of Medicine, Northwestern University, Chicago, IL, USA; h Department of Health Outcomes & Behavior, H. Lee Moffitt Cancer Center, Tampa, Florida, USA; i Department of Surgery, Division of Public Health Sciences, Washington University School of Medicine, St. Louis, MO, USA

**Keywords:** HINTS data, Genetic testing, cancer history, Gender, Rural-urban status, Physical activity, Obesity

## Abstract

**Objective:**

Genetic testing and precision medicine enhance early detection and personalized treatment, potentially reducing morbidity and mortality. We examined how beliefs and perceptions of genetic testing and precision medicine differ by cancer history, gender, rural-urban status, physical activity, and obesity.

**Methods:**

We analyzed 3865 respondents from the Health Information National Trends Survey (HINTS) 5, Cycle 4, administered February 2020–June 2020. We assessed the associations between cancer history, demographic and health-related factors, with knowledge, beliefs, and perceptions of genetic testing and precision medicine. Adjusted Odds Ratios (AORs) and 95 % Confidence Intervals (CIs) were calculated.

**Results:**

Cancer survivors were more likely to have had genetic testing (AOR = 1.61; 95 % CI, 1.03–2.53) and to want to know if a genetic change increases cancer risk (AOR = 1.65; 95 % CI, 1.12–2.44). Men were more likely to report knowledge of precision medicine (AOR = 1.52; 95 % CI, 1.16–1.98) but less likely to believe genetic information is important for cancer prevention (AOR = 0.72; 95 % CI, 0.55–0.94). Those with less physical activity were less likely to report knowledge of precision medicine (AOR = 0.67; 95 % CI, 0.47–0.96) and genetic tests (AOR = 0.70; 95 % CI, 0.51–0.97). Individuals with obesity were more likely to believe genetic information is important for preventing (AOR = 1.36; 95 % CI, 1.06–1.75) and treating cancer (AOR = 1.27; 95 % CI, 1.02–1.59).

**Conclusion:**

Knowledge and beliefs about genetic testing and precision medicine vary by sociodemographic factors, in particular individuals with obesity are more likely to value genetic information.

## Highlights

• Cancer survivors value knowing if a genetic change increases their cancer risk.
• Men are less likely to value genetic information for cancer prevention.
• Lower physical activity is associated with less knowledge of genetic tests.
• Urban residents were more aware of genetic tests than rural residents.
• Individuals with obesity valued genetic info for cancer prevention and treatment.

## 1 Introduction

Rapid technical developments resulting from the Human Genome Project have increased our understanding of genetic factors and their impact on human life and diseases, including cancer, dementia, and diabetes (Roses, 2003; Bailey et al., 2014; Roychowdhury and Chinnaiyan, 2014). Genetic testing, combined with precision medicine—defined as tailored or personalized disease management that accounts for an individual's genetic, environmental, and lifestyle characteristics—can improve diagnostic accuracy as a personalized approach to prevent and treat diseases (Evans et al., 2024). Hundreds of genetic tests are available to estimate cancer risk, including multi-cancer detection tests (Phillips et al., 2018).

Some known barriers to genetic testing include 1) perceived cost; 2) knowledge and beliefs about the process; 3) fear related to having increased cancer risk, genetic discrimination, implications of receiving a positive test result; 4) inconsistent and complex insurance coverage policies; 5) distrust in the medical system; and 6) a lack of actionable solutions if a genetic predisposition is identified (Guo et al., 2022; Blomen et al., 2021; National Academies of Sciences, 2018; Dusic et al., 2022). As a result, it is crucial to recognize differences in perspectives on genetic testing, for which some studies have started to evaluate global awareness and attitudes (Eum et al., 2018; Dusic et al., 2022). Such evaluation can inform health messaging for population screening, as the prevalence and number of genetic tests and precision medicine approaches continue to grow (Makhnoon et al., 2022).

Use and knowledge of genetic testing or precision medicine may be influenced by clinical (i.e., cancer survivorship status), demographic (gender, rural/urban status), and health factors (obesity, physical activity) (Salloum et al., 2018; Hong et al., 2024; Hong et al., 2024; Tiner et al., 2022; Swoboda et al., 2023; Thakker et al., 2023; Dong et al., 2022). For instance, cancer survivors have reported a willingness to undergo predictive and prognostic genetic testing, but report being more hesitant to receive results that increase worry about conditions they may never develop (Gray et al., 2012). Women are more likely than men to report undergoing genetic testing (Swoboda et al., 2023; Tiner et al., 2022). Urban residents report greater awareness of testing than rural residents (Salloum et al., 2018 (Swoboda et al., 2023; Tiner et al., 2022)—who have less access to testing (Gardner et al., 2021). Obesity and physical activity are known cancer risk factors (Lauby-Secretan et al., 2016; McTiernan et al., 2019); however, individuals with obesity are less likely to use genetic testing for cancer risk compared to individuals who are not obese (Hong et al., 2024). Obesity and being sedentary are also associated with lower cancer screening uptake (Unanue-Arza et al., 2021). Understanding, awareness, and perception of genetic testing by clinical, demographic, and health behaviors are important for removing barriers and may help guide patient-provider communication by exposing knowledge gaps and concerns. Although other contextual factors exist, we aimed to examine clinical, demographic, and health factors that can influence awareness of genetic tests, sharing of genetic results, and beliefs and perceptions regarding the importance of genetic testing to prevent, detect, and treat cancer.

## 2 Methods

### 2.1 Study design and data source

We performed cross-sectional analysis using data from the Health Information National Trends Survey (HINTS 5, Cycle 4), administered February 2020–June 2020 (HINTS 5, 2020a). HINTS is a nationally representative estimate of noninstitutionalized adults (>18 years) living in the US. The survey was centered on health-related knowledge and beliefs, including attitudes about cancer related to early detection, prevention, treatment, and genetic testing. Sampling involved a two-stage design that included a sample of addresses, followed by a selection of one adult from each sampled household to be representative of the entire US population. The survey targeted 10,531 individuals with a 36.7 % response rate, resulting in 3865 respondents. This study was exempt from institutional review board approval due to the use of publicly available, de-identified data.

### 2.2 Outcomes

Our outcomes of interest were the utilization, awareness, beliefs, and perceptions of genetic testing and precision medicine. Survey questions can be found in the HINTS 5 Cycle 4 survey 2020 (HINTS 5, 2020b). Outcome details and response dichotomization are explained below.

***Precision Medicine:***

Respondents were asked: “Precision medicine is an approach for disease treatment and prevention that accounts for individual differences in genes, environment, and lifestyle. Before completing this survey, had you ever heard of approaches like precision medicine?” Responses included “yes” and “no.”

***Genetic Testing Knowledge, Utilization, and Result Sharing:***

*Genetic Tests Awareness:* Respondents were asked, “Which of the following types of genetic tests have you heard of? Mark all that apply.” We dichotomized responses into 1) heard of any genetic testing (i.e., ancestry testing, genetic health risk testing, high-risk cancer testing, other) vs. 2) not heard of genetic testing (not sure or none selected).

*Genetic Tests Utilized:* Respondents who indicated awareness of genetic testing were asked, “Have you ever had any of the following types of genetic tests? Mark all that apply.” Responses were dichotomized into 1) had any genetic testing (i.e., ancestry testing, genetic health risk testing, high-risk cancer testing, or other) vs. 2) did not have genetic testing (not sure or none of the above).

*Understanding Genetic Test Results:* Respondents were asked, “If you had a genetic test, who helped you understand the results? Mark all that apply.” We dichotomized responses into 1) those who had someone help them understand their results (i.e., health care provider, genetic counselor, spouse/partner, parents, siblings, children, friend, or other) vs. 2) those who responded that no one helped.

*Sharing Genetic Test Results:* Respondents were asked, “If you had a genetic test, who did you share the results with? Mark all that apply.” We combined and created a binary variable: if the respondents said yes to any of the choices (i.e., health care provider, genetic counselor, spouse/partner, parents, siblings, children, friend, other, and did not share the results) vs. if they did not share the results with anyone.

To maximize power for multivariable modeling, responses for genetic testing awareness, utilization, and sharing were dichotomized into any awareness/utilization/sharing vs. none.

***Beliefs about Genetic Testing and Cancer:***

*Prevent, Detect, and Treat Cancer:* Respondents were asked: “How important is knowing a person's genetic information for [preventing/detecting/treating] cancer?” Responses (i.e., not at all, a little, somewhat, and very) were dichotomized into “very” vs. “somewhat/a little/not at all.”

*Behavior Change:* Respondents were asked, “How much do you agree or disagree with the following statement? If I found out from a genetic test that I was at substantial risk of cancer, I would change my behaviors such as diet, exercise, and getting routine medical tests…” Responses (i.e., a lot, somewhat, a little, and not at all) were dichotomized into those who responded with “a lot” vs. those who responded “somewhat/a little/not at all.”

*Cancer Risk:* Respondents were asked, “How much would you want to know if you have a genetic change that increases your chances of getting cancer?” Responses (i.e., strongly agree, somewhat agree, strongly disagree, and somewhat disagree) were dichotomized into “strongly agree” vs. “somewhat agree/strongly disagree/somewhat disagree.”

### 2.3 Exposures

Our exposures of interest included clinical (i.e., history of cancer), demographic (gender, rural-urban status), and health (obesity, physical activity) characteristics.

***Clinical characteristics:*** Self-reported history of cancer was evaluated by asking, “Have.

you ever been diagnosed as having cancer?”. Individuals who reported a history of cancer were defined as cancer survivors.

***Demographic characteristics:*** Self-reported gender was categorized as men vs. women.

Residence was categorized as urban vs. rural, with Rural-Urban Continuum codes (urban: 1–3, rural: ≥4) (Bhavsar et al., 2022).

***Health characteristics:*** Respondents were asked, “In a typical week, how many days do you do any physical activity or exercise of at least moderate intensity, such as brisk walking, bicycling at a regular pace, and swimming at a regular pace (do not include weightlifting)?” We created a binary variable with those reporting <3 days per week of physical activity vs. those reporting ≥3 days per week. Additionally, the Body Mass Index (BMI) variable was created using standard cut points and categorized as individuals living with obesity (>30 kg/m2) and individuals not living with obesity (<30 kg/m2) (“CDC, 2024”).

### 2.4 Confounders

We selected covariates based on known confounders, including age at the time of survey (≤64 vs. >65), gender (men vs. women), race (White vs. non-White), and ethnicity (Hispanic vs. non-Hispanic) (Swoboda et al., 2023; Makhnoon et al., 2022).

### 2.5 Statistical analysis

We analyzed data using survey weights based on population estimates from the American Community Survey to account for non-response and coverage errors, making the results generalizable to the US population based on HINTS protocols (HINTS 5, 2020a). We used weighted chi-square tests for categorical variables to compare differences in our population by cancer history, gender, urban-rural residence, physical activity, and obesity status. We used weighted multivariable logistic regression models to assess the association between predictors of (1) cancer history, (2) gender, (3) urban and rural residence, (4) physical activity, and (5) obesity. Outcomes included awareness, perception, and use of genetic testing and precision medicine. Each outcome and exposure was modeled separately. Each exposure was modeled separately based on our a priori objective to examine each clinical, demographic, and health factor as a separate primary exposure and to minimize any collinearity between exposures. Models were adjusted for known confounders: age, gender, race, and ethnicity. Gender was assessed as a primary exposure and included as a covariate in models evaluating other exposures (i.e., cancer history, rural/urban status, physical activity, and obesity). Significance was set at *p* ≤ 0.05, and complete-case analysis was used for all analyses. We used SAS 9.4 (SAS Institute, Cary, NC) for all analyses.

## 3 Results

### 3.1 Population characteristics

Of the 3865 respondents, 51 % were women, 68.2 % of respondents were > 50 years old, 87.8 % lived in an urban area, 30.3 % were college graduates, 78.7 % were White, 83 % were non-Hispanic, and 57.5 % earned an annual household income of <$75,000 **(**Table 1**)**. Out of the total respondents, 9.2 % had a history of cancer, 33 % were living with obesity, and 55.1 % participated in physical activity >3 days a week. **Supplemental Tables 1–4** (Additional file 1) describe the cohort characteristics stratified by cancer history, gender, urban-rural status, physical activity, and obesity.

**Table 1:** Demographic characteristics of 3865 U.S. adults — Health Information National Trends Survey 5, Cycle 4 (February 2020–January 2020).

Characteristics	Total
	N (%)⁎
Gender	
Male	1487 (49.0)
Female	2052 (51.0)
Missing	326

Age	
18–34	484 (26.2)
35–49	703 (25.5)
50–64	1142 (27.7)
65–74	869 (12.0)
75+	540 (8.6)
Missing	127

Rural Urban Status	
Urban	3435 (87.8)
Rural	430 (12.2)

Highest level of school completed	
College Graduate	1663 (30.3)
Not College Graduate	2059 (69.7)
Missing	143

White	
Yes	2707 (78.7)
No	867 (21.3)
Missing	291

Ethnicity	
Non-Hispanic	2894 (83.0)
Hispanic	596 (17.0)
Missing	37

Combined annual household income#	
Less than or equal to 75,000	2127 (57.5)
More than 75,000	1321 (42.5)
Missing	41

Ever Had Cancer	
Yes	626 (9.2)
No	3168 (90.8)
Missing	71

Obesity status	
Individuals living with obesity	1237 (33.0)
Individuals without obesity	2508 (67.0)
Missing	120

Physical Activity	
Three of more days a week	2069 (55.1)
Less than three days a week	1729 (44.9)
Missing	67

Frequency calculated among respondents with non-missing data.

⁎ Presented as row weighted percentage.

# Combined annual household income is in U.S. dollars.

### 3.2 Association by clinical characteristics

There was no difference in knowledge of precision medicine (AOR = 1.00; 95 % CI, 0.66–1.53) or genetic tests (AOR = 0.96; 95 % CI, 0.67–1.40) between cancer survivors and those without a cancer history after adjusting for age, gender, race, and ethnicity **(**Table 2**)**. Cancer survivors (*n* = 626) were more likely (AOR = 1.61; 95 % CI, 1.03–2.53) to have engaged in genetic testing compared to those without a cancer history. We observed no differences between groups in having someone help with understanding genetic test results or with sharing test results.

**Table 2:** Odds Ratios and 95 % Confidence Intervals for Associations between Awareness, Perception and Uptake of Genetic Testing and Precision Medicine by Clinical Characteristics (History of Cancer) –Health Information National Trends Survey 5, Cycle 4 (February 2020–January 2020; U.S.)

Outcomes	Cancer Survivors	No Cancer History	Unadjusted OR	Adjusted OR^h^
	N (%)⁎	N (%)⁎	OR (95 % CI)	OR (95 % CI)
Heard of approaches like precision medicine				
Yes	102 (14.5)	492 (13.9)	1.05 (0.71–1.55)	1.00 (0.66–1.53)
No	507 (85.5)	2627 (86.1)	1.00	1.00

Heard of genetic tests				
Heard of genetic tests a	446 (75.8)	2339 (77.0)	0.94 (0.69–1.28)	0.96 (0.67–1.40)
Not heard of genetic tests b	146 (24.2)	727 (23.0)	1.00	1.00

Had genetic tests				
Had genetic tests c	160 (32.7)	562 (20.6)	1.87 (1.27–2.75)	1.61 (1.03–2.53)
Did not have genetic tests d	313 (67.3)	1877 (79.4)	1.00	1.00

Someone helped understand the genetic test results				
Someone helped e	87 (63.9)	249 (49.1)	1.83 (1.10–3.07)	1.66 (0.94–2.95)
No one helped	68 (36.1)	280 (50.9)	1.00	1.00

Shared genetic tests results with someone				
Shared f	137 (86.2)	461 (85.3)	1.08 (0.44–2.65)	0.93 (0.32–2.66)
Not shared	19 (13.8)	87 (14.7)	1.00	1.00

Importance of knowing a person's genetic information for preventing cancer				
Very	242 (41.0)	1434 (50.7)	0.68 (0.47–0.98)	0.82 (0.55–1.24)
Somewhat + A little + Not at all	353 (59.0)	1585 (49.3)	1.00	1.00

Importance of knowing a person's genetic information for detecting cancer early				
Very	292 (51.2)	1655 (57.7)	0.77 (0.54–1.09)	0.99 (0.68–1.45)
Somewhat + A little + Not at all	303 (48.8)	1360 (42.3)	1.00	1.00

Importance of knowing a person's genetic information for treating cancer				
Very	270 (46.2)	1445 (50.3)	0.85 (0.60–1.22)	1.04 (0.69–1.56)
Somewhat + A little + Not at all	322 (53.8)	1549 (49.7)	1.00	1.00

Importance of knowing that a genetic change increases your chance of getting cancer				
A lot	345 (58.4)	1530 (48.6)	1.49 (1.11–1.99)	1.65 (1.12–2.44)
Somewhat + A little + Not at all	267 (41.6)	1608 (51.4)	1.00	1.00

Change behaviors such as diet, exercise and getting routine medical tests if a genetic test found them at high risk of cancer				
Strongly agree	312 (51.9)	1593 (51.1)	1.03 (0.77–1.38)	1.24 (0.88–1.76)
Somewhat agree+ Somewhat Disagree +Strongly disagree g	297 (48.1)	1508 (48.9)	1.00	1.00

1.00 refers to referent group for outcome. No history of cancer was referent group for exposure.

a The “heard genetic tests” category included respondents who heard of ancestry testing, multiple genetic tests, genetic health risk testing, high risk cancer testing and other testing.

b Respondents who were not sure or have heard none of the genetic tests were categorized as “not heard”.

c The “had genetic tests” category included respondents who had ancestry testing, multiple genetic tests, genetic health risk testing, high risk cancer testing and other testing.

d Respondents who did were not sure or chose none of the genetic tests were categorized as “did not have”.

e The “someone helped” category included respondents who received help to understand genetic test results from health care provider, genetic counselor, spouse, parents, siblings, children, friend, multiple genetic testing selected and others.

f The “shared” category included respondents who shared genetic test results with health care provider, genetic counselor, spouse, parents, siblings, children, friend, multiple genetic testing selected and others.

g This category includes those respondents who somewhat agree, somewhat disagree, and strongly disagree.

h Odds ratios were adjusted for age, gender, race, and ethnicity.

⁎ Presented as row weighted percentage.

**Table 3:** Odds Ratios and 95 % Confidence Intervals for Associations between Awareness, Perception and Uptake of Genetic Testing and Precision Medicine by Demographic Characteristics (Gender and Rural-Urban Status)-- Health Information National Trends Survey 5, Cycle 4 (February 2020–January 2020; U.S.)

Outcomes	Male	Female	Unadjusted OR	Adjusted OR^i^	Urban	Rural	Unadjusted OR	Adjusted OR^h^
	N (%)*	N (%)*	OR (95 % CI)	OR (95 % CI)	N (%)*	N (%)*	OR (95 % CI)	OR (95 % CI)

Heard of approaches like precision medicine								
Yes	264 (16.2)	300 (11.9)	1.43 (1.13–1.83)	1.52 (1.16–1.98)	548 (14.4)	55 (11.7)	1.27 (0.79–2.05)	1.53 (0.94–2.51)
No	1203 (83.8)	1717 (88.1)	1.00	1.00	2798 (85.6)	366 (88.3)	1.00	1.00

Heard of genetic tests								
Heard genetic tests a	1089 (76.3)	1545 (78.9)	0.86 (0.62–1.19)	0.81 (0.57–1.15)	2516 (77.2)	304 (74.6)	1.15 (0.74–1.79)	1.77 (1.04–3.01)
Not heard of genetic tests b	356 (23.7)	428 (21.1)	1.00	1.00	779 (22.8)	110 (25.4)	1.00	1.00

Had genetic tests								
Had genetic tests c	245 (16.5)	431 (26.4)	0.55 (0.41–0.73)	0.54 (0.40–0.73)	657 (21.8)	70 (20.7)	1.06 (0.68–1.65)	1.22 (0.75–1.98)
Did not have genetic tests d	881 (83.5)	1192 (73.6)	1.00	1.00	1978 (78.2)	242 (79.3)	1.00	1.00

Someone helped understanding the genetic test results								
Someone helped e	103 (45.6)	212 (54.9)	0.69 (0.43–1.12)	0.67 (0.42–1.07)	306 (50.5)	32 (54.5)	0.85 (0.41–1.76)	0.88 (0.41–1.91)
No one helped	122 (54.4)	203 (45.1)	1.00	1.00	318 (49.5)	33 (45.5)	1.00	1.00

Shared genetic test results with someone								
Shared f	191 (82.0)	368 (87.3)	0.66 (0.32–1.39)	0.62 (0.27–1.42)	546 (85.0)	57 (88.1)	0.76 (0.30–1.90)	1.02 (0.36–2.87)
Not shared	37 (18.0)	62 (12.7)	1.00	1.00	99 (15.0)	8 (11.9)	1.00	1.00

Importance of knowing a person's genetic information for preventing cancer								
Very	586 (46.2)	992 (53.0)	0.76 (0.61–0.95)	0.72 (0.55–0.94)	1533 (51.2)	167 (38.1)	1.71 (1.20–2.45)	1.50 (0.99–2.28)
Somewhat + A little + Not at all	836 (53.8)	973 (47.0)	1.00	1.00	1716 (48.8)	244 (61.9)	1.00	1.00

Importance of knowing a person's genetic information for detecting cancer early								
Very	694 (54.2)	1143 (60.1)	0.79 (0.62–1.00)	0.76 (0.57–1.00)	1777 (58.5)	197 (46.9)	1.60 (1.15–2.20)	1.49 (0.99–2.23)
Somewhat + A little + Not at all	731 (45.8)	817 (39.9)	1.00	1.00	1470 (41.5)	212 (53.1)	1.00	1.00

Importance of knowing a person's genetic information for treating cancer?								
Very	613 (48.7)	997 (51.3)	0.90 (0.72–1.13)	1.17 (0.90–1.51)	1553 (51.0)	187 (40.8)	1. 51 (1.09–2.10)	1.31 (0.89–1.92)
Somewhat + A little + Not at all	796 (51.3)	957 (48.7)	1.00	1.00	1668 (49.0)	222 (59.2)	1.00	1.00

Importance of knowing that a genetic change increases your chance of getting cancer								
A lot	690 (48.5)	1059 (50.2)	0.94 (0.76–1.15)	0.88 (0.71–1.09)	1675 (50.1)	202 (45.3)	1.21 (0.85–1.73)	1.14 (0.77–1.68)
Somewhat + A little + Not at all	767 (51.5)	955 (49.8)	1.00	1.00	1664 (49.9)	212 (54.7)	1.00	1.00

Would change behaviors such as diet, exercise and getting routine medical tests if a genetic tests if found them at high risk of cancer								
Strongly agree	673 (47.2)	1114 (54.3)	0.75 (0.59–0.96)	0.69 (0.53–0.89)	1727 (51.5)	196 (48.9)	1.11 (0.78–1.58)	0.88 (0.56–1.37)
Somewhat agree + Somewhat dis- agree+ strongly disagree^g^	789 (52.8)	899 (45.7)	1.00	1.00	1603 (48.5)	223 (51.1)	1.00	1.00

1.00 refers to referent group for outcome. *Presented as row weighted percentage.

a The “heard genetic tests” category included respondents who heard of ancestry testing, multiple genetic tests, genetic health risk testing, high risk cancer testing and other testing.

b Respondents who were not sure or have heard none of the genetic tests were categorized as “not heard”.

c The “had genetic tests” category included respondents who had ancestry testing, multiple genetic tests, genetic health risk testing, high risk cancer testing and other testing.

d Respondents who did were not sure or chose none of the genetic tests were categorized as “did not have”.

e The “someone helped” category included respondents who received help to understand genetic test results from health care provider, genetic counselor, spouse, parents, siblings, children, friend, multiple genetic testing selected and others.

f The “shared” category included respondents who shared genetic test results with health care provider, genetic counselor, spouse, parents, siblings, children, friend, multiple genetic testing selected and others.

g The category includes those respondents who somewhat agree, somewhat disagree and strongly disagree.

h Odds ratios were adjusted for age, gender, race, and ethnicity.

i Odds ratios were adjusted for age, race, and ethnicity.

**Table 4:** Odds Ratios and 95 % Confidence Intervals for Associations between Awareness, Perception and Uptake of Genetic Testing and Precision Medicine by Health Characteristics (Physical Activity and Obesity)-- Health Information National Trends Survey 5, Cycle 4 (February 2020–January 2020; U.S.)

Outcomes	Physical activity < 3 days	Physical activity ≥ 3 days	Unadjusted OR	Adjusted OR^h^	Individuals living with obesity	Individuals without obesity	Unadjusted OR	Adjusted OR^h^
	N (%)*	N (%)*	OR (95 % CI)	OR (95 % CI)	N (%)*	N (%)*	OR (95 % CI)	OR (95 % CI)
Heard of approaches like precision medicine								
Yes	216 (11.1)	384 (16.7)	0.62 (0.45–0.87)	0.67 (0.47–0.96)	162 (10.7)	426 (15.8)	0.64 (0.44–0.92)	0.65 (0.44–0.95)
No	1477 (88.9)	1650 (83.3)	1.00	1.00	1056 (89.3)	2030 (84.2)	1.00	1.00

Heard of genetic tests								
Heard genetic tests a	1192 (73.5)	1602 (80.2)	0.68 (0.53–0.89)	0.70 (0.51–0.97)	926 (79.2)	1834 (76.1)	1.20 (0.94–1.52)	1.14 (0.87–1.51)
Not heard of genetic tests b	475 (26.5)	395 (19.8)	1.00	1.00	281 (20.8)	571 (23.9)	1.00	1.00

Had genetic tests								
Had Genetic tests c	273 (19.5)	448 (23.4)	0.79 (0.60–1.05)	0.86 (0.63–1.16)	228 (17.8)	488 (24.0)	0.69 (0.54–0.88)	0.74 (0.56–0.96)
Did not have genetic tests d	981(80.5)	1215 (76.6)	1.00	1.00	733 (82.2)	1428 (76.0)	1.00	1.00

Someone helped understanding the genetic test								
Someone helped e	140 (49.7)	193 (52.1)	0.91 (0.56–1.48)	0.87 (0.50–1.52)	107 (49.0)	223 (52.4)	0.87 (0.55–1.38)	0.78 (0.47–1.29)
No one helped	143 (50.3)	207 (47.9)	1.00	1.00	109 (51.0)	232 (47.6)	1.00	1.00

Shared genetic test results with someone								
Shared f	241(83.5)	355 (87.2)	0.74 (0.37–1.51)	0.64 (0.28–1.47)	190 (86.2)	401 (86.5)	0.98 (0.45–2.12)	0.95 (0.39–2.29)
Not shared	49 (16.5)	57 (12.8)	1.00	1.00	31 (13.8)	69 (13.5)	1.00	1.00

Importance of knowing a person's genetic information for preventing cancer								
Very	767 (51.5)	913 (48.0)	1.15 (0.91–1.45)	1.00 (0.78–1.28)	604 (54.5)	1053 (47.0)	1.35 (1.07–1.72)	1.36 (1.06–1.75)
Somewhat + A little + Not at all	868 (48.5)	1073 (52.0)	1.00	1.00	588 (45.5)	1326 (53.0)	1.00	1.00

Importance of knowing a person's genetic information for detecting cancer early								
Very	880 (58.6)	1071 (55.8)	1.12 (0.87–1.44)	1.05 (0.80–1.36)	659 (58.7)	1266 (56.1)	1.11 (0.89–1.39)	1.12 (0.87–1.44)
Somewhat + A little + Not at all	756 (41.4)	906 (44.2)	1.00	1.00	527 (41.3)	1115 (43.9)	1.00	1.00

Importance of knowing a person's genetic information for treating cancer								
Very	792 (52.1)	930 (47.9)	1.18 (0.91–1.53)	1.10 (0.84–1.45)	607 (53.5)	1091(47.9)	1.25 (1.01–1.56)	1.27 (1.02–1.59)
Somewhat + A little + Not at all	830 (47.9)	1039 (52.1)	1.00	1.00	574 (46.5)	1271 (52.1)	1.00	1.00

Importance of knowing that a genetic change that increases your chance of getting cancer								
A lot	835 (49.8)	1017 (48.9)	1.04 (0.82–1.32)	0.99 (0.76–1.29)	639 (50.6)	1197 (49.2)	1.06 (0.85–1.32)	1.08 (0.86–1.35)
Somewhat + A little + Not at all	852 (50.2)	1006 (51.1)	1.00	1.00	580 (49.4)	1244 (50.8)	1.00	1.00

Would change behaviors such as diet, exercise and getting routine medical tests if a genetic test found them at high risk of cancer								
Strongly agree	811 (47.9)	1096 (53.6)	0.80 (0.62–1.03)	0.70 (0.54–0.92)	614 (51.1)	1274 (51.4)	1.00 (0.75–1.31)	1.02 (0.76–1.36)
Somewhat agree + Somewhat dis- agree+ strongly disagree^g^	869 (52.1)	931 (46.4)	1.00	1.00	598 (48.9)	1173 (48.6)	1.00	1.00

1.00 refers to referent group for outcome. *Presented as row weighted percentage.

a The “heard genetic tests” category included respondents who heard of ancestry testing, multiple genetic tests, genetic health risk testing, high risk cancer testing and other testing.

b Respondents who were not sure or have heard none of the genetic tests were categorized as “not heard”.

c The “had genetic tests” category included respondents who had ancestry testing, multiple genetic tests, genetic health risk testing, high risk cancer testing and other testing.

d Respondents who did were not sure or chose none of the genetic tests were categorized as “did not have”.

e The “someone helped” category included respondents who received help to understand genetic test results from health care provider, genetic counselor, spouse, parents, siblings, children, friend, multiple genetic testing selected and others.

f The “shared” category included respondents who shared genetic test results with health care provider, genetic counselor, spouse, parents, siblings, children, friend, multiple genetic testing selected and others.

g The category includes those respondents who somewhat agree, somewhat disagree and strongly disagree.

h Odds ratios were adjusted for age, gender, race, and ethnicity.

Both groups had similar beliefs regarding the importance of genetic information for cancer prevention (AOR = 0.82; 95 % CI, 0.55–1.24), detection (AOR = 0.99; 95 % CI, 0.68–1.45), and treatment (AOR = 1.04; 95 % CI, 0.69–1.56). Cancer survivors were more likely (AOR = 1.65; 95 % CI, 1.12–2.44) to want to know if a genetic change increased their cancer risk. However, they were no more likely to change their behavior (i.e., diet, exercise, routine medical tests) if a genetic test indicated high cancer risk.

### 3.3 Association by demographic characteristics

Men were 52 % (AOR = 1.52; 95 % CI, 1.16–1.98) more likely to report knowledge of approaches like precision medicine compared to women **(**Table 3**).** Awareness of genetic testing was similar between men and women. Men were 45 % (AOR = 0.54; 95 % CI, 0.40–0.73) less likely to report having had any genetic test and 28 % and 24 % less likely to believe genetic tests were important for cancer prevention (AOR = 0.72; 95 % CI, 0.55–0.94) and early detection (AOR = 0.76; 95 % CI, 0.57–1.00), respectively. Moreover, men were 31 % (AOR = 0.69; 95 % CI, 0.53–0.89) less likely to change their behavior if a genetic test indicated they had substantial cancer risk. Men and women were similar in regard to sharing results or having someone help with understanding results.

There was no difference in knowledge of precision medicine (AOR = 1.53; 95 % CI, 0.94–2.51) between urban and rural residents; however, urban residents were 77 % more likely (AOR = 1.77; 95 % CI, 1.04–3.01) to know about genetic tests. No differences were observed across groups in sharing genetic test results or having someone help understand results. In unadjusted analyses, urban residents were more likely to report that genetic testing was important for cancer prevention, detection, and treatment. After adjustment, findings were similar but not statistically significant for prevention (AOR = 1.50; 95 % CI, 0.99–2.28), early detection (AOR = 1.49; 95 % CI, 0.99–2.23), and treatment (AOR = 1.31; 95 % CI, 0.89–1.92). Urban residents were no more likely to report they would change behaviors (AOR = 0.88; 95 % CI, 0.56–1.37) if a genetic test indicated high cancer risk.

### 3.4 Association by health characteristics

Individuals who performed physical activity <3 days a week were 33 % (AOR = 0.67; 95 % CI, 0.47–0.96) less likely to know about precision medicine and 30 % (AOR = 0.70; 95 % CI, 0.51–0.97) less likely to know about genetic tests, compared to those exercising >3 days a week **(**Table 4**)**. No difference was observed in having had a genetic test, sharing test results, having someone help understand, or in the perceived importance of genetic tests for cancer prevention, detection, or treatment across physical activity groups. Those with less physical activity were 30 % (AOR = 0.70; 95 % CI, 0.54–0.92) less likely to change behaviors (e.g., diet, exercise, and routine medical testing) if a genetic test indicated high cancer risk.

Individuals with obesity were 35 % less likely to know about precision medicine (AOR = 0.65; 95 % CI, 0.44–0.95) and 26 % less likely to have received genetic testing (AOR = 0.74; 95 % CI, 0.56–0.96). There was no difference in genetic testing knowledge, having someone help understand genetic tests, and sharing of genetic test results between those with and without obesity. Individuals with obesity were 36 % and 27 % more likely to value testing for cancer prevention (AOR = 1.36; 95 % CI, 1.06–1.75) and treatment (AOR = 1.27; 95 % CI, 1.02–1.59), respectively, compared to those without obesity. Both groups had similar beliefs about changing behavior (AOR = 1.02; 95 % CI, 0.76–1.36) if a test indicated high cancer risk.

## 4 Discussion

We examined how clinical, demographic, and health factors are associated with awareness, beliefs, perception, and use of genetic testing and precision medicine. Our multifactorial approach comprehensively explains how numerous factors influence attitudes and behaviors toward genetic testing. Although prior studies examined the influence of demographic and clinical factors on awareness and uptake of genetic testing (Makhnoon et al., 2022; Tiner et al., 2022; Thakker et al., 2023), we provide additional insights into how lifestyle factors may impact knowledge and receptiveness of testing for cancer prevention, detection, and treatment. As genetic tests expand, understanding public perceptions is crucial to ensure equitable uptake and minimize disparities.

Cancer survivors were more likely to report receiving genetic tests, consistent with previous findings (Makhnoon et al., 2022; Thakker et al., 2023; Swoboda et al., 2023; Dong et al., 2022; Tiner et al., 2022). Cancer survivors and those without a cancer history were equally likely to report knowledge of precision medicine and genetic testing. Thakker et al. provided descriptive data on beliefs of genetic testing among HINTS respondents with breast/ovarian and prostate cancer, and observed a lower awareness of testing among patients with prostate cancer (Thakker et al., 2023). We found that both cancer survivors (14.5 %) and those without a cancer history (13.9 %) had limited knowledge of precision medicine. We found no difference between groups on perceived importance of genetic information for cancer prevention, detection, or treatment. However, cancer survivors were 65 % more likely to want to know if a genetic change increased their risk, although they were no more likely to change behaviors if a genetic test revealed higher risk—potentially because genetic changes do not necessarily indicate a cancer diagnosis will occur. For instance, women who carry BRCA1 and BRCA2 gene mutations are at high risk of various cancers (e.g., breast, pancreas, and ovarian cancers) (Li et al., 2022)—but not everyone who inherits these mutations will develop cancer.

The increased genetic testing among women we observed aligns with previous studies (Makhnoon et al., 2022; Swoboda et al., 2023; Roberts et al., 2019; Childers et al., 2018; Hong et al., 2023; Hamilton et al., 2015; Mai et al., 2014). This is likely because a large number of genetic tests are provided for breast/ovarian cancers (Makhnoon et al., 2022), and women generally have more interest in health topics and are more inclined toward health-seeking behaviors (Ek, 2015; Leppin and Nielsen, 2022; Thompson et al., 2016). Men, however, can be reluctant to get tested and may express dislike when notified of their risk. Men may also delay seeking care for various reasons: downplaying their risk, workplace obligations, and potentially considering health promotion and prevention as unacceptable masculine behavior (Giorgianni Giorgianni Jr et al., 2013; Novak et al., 2019; Yousaf et al., 2015). Our results highlight the need for increased outreach to men regarding genetic testing.

Urban residents are more aware of genetic tests than rural residents, consistent with previous findings (Salloum et al., 2018; Apathy et al., 2018). Lower awareness in rural areas can be due to reduced service availability and limited access to providers and facilities that offer genetic testing and counseling, which are typically concentrated in urban academic centers (Gardner et al., 2021). Rural residents can also experience reduced access to resources such as financial counselors and mental health professionals who help patients navigate unmet supportive care needs (Asante et al., 2023). To access these services, rural residents may have to travel long distances to urban centers, resulting in further financial, social, and physical burdens (Kruse-Diehr et al., 2022; Tzelepis et al., 2018). Rural residents may also face challenges in prioritizing these supportive services (Lewis-Thames et al., 2023), leading to less health service utilization. This is reflected in urban residents' beliefs about the importance of genetic testing. In *unadjusted analyses*, urban residents were 71 %, 60 %, and 51 % more likely to report that genetic tests were important for preventing, detecting, and treating cancer, respectively. While these associations did not remain statistically significant after adjustment, these findings, coupled with the difference in awareness of genetic tests, suggest there are opportunities for focused genetic testing programs and education in rural areas. Increasing genetic literacy may be one way to increase awareness of genetic testing benefits (Little et al., 2022).

Respondents with less physical activity were less likely to know about precision medicine and genetic testing, and to have had a genetic test—potentially due to a lower health focus among those who exercise less. While physical activity was not linked to beliefs about the importance of genetic testing for cancer, those reporting less physical activity were 30 % less likely to agree they would change their behavior if found to have increased cancer risk. Behavior change evidence following a genetic test is inconsistent (Horne et al., 2018). Given the more immediate, well-established benefits of physical activity for a wide range of health outcomes (e.g., cardiovascular health, obesity, diabetes) (Cleven et al., 2020), a distant cancer risk indicated by a genetic test might not be motivating enough to change behavior.

Previous studies have focused on genetic determinants of obesity (Masood and Moorthy, 2023; Concepción-Zavaleta et al., 2024) and genetic testing uptake (Hong et al., 2023) among individuals with obesity. However, to our knowledge, we are one of the first studies to explore beliefs and perceptions of the importance of genetic testing by obesity status and physical activity. We found that individuals with obesity reported less knowledge of precision medicine and use of genetic tests, aligning with literature reporting decreased uptake of testing for cancer risk among individuals with obesity (Hong et al., 2023). However, we additionally observed that individuals living with obesity were 36 % and 27 % more likely to believe testing was important for preventing and treating cancer, respectively. Individuals living with obesity may view it as important because they are at increased risk of certain cancers and other diseases.

Study strengths include 1) using a large, nationally representative sample of US adults and 2) the ability to go beyond genetic testing awareness and uptake to comprehensively examine beliefs regarding the importance of genetic testing for cancer prevention, treatment, and detection across clinical, demographic, and health factors. However, our study is limited by self-reported data that may introduce bias. Additionally, as HINTS is a cross-sectional study, we cannot establish temporality between our exposures and outcomes. Furthermore, HINTS survey items grouped distinct genetic testing types under broader categories that we dichotomized (e.g., ancestry testing, genetic health risk testing, high-risk cancer testing, etc., vs none) for analysis—potentially masking meaningful differences as the combined approach was designed to maximize power for our study.

Our results highlighted that while knowledge of genetic testing is high (76.9 %), knowledge of precision medicine remains low (14.1 %), independent of clinical, demographic, and health factors. This could be partly attributed to provider-patient discussions on precision medicine being uncommon. Future work should enhance understanding of genetic testing and precision medicine benefits. Public awareness campaigns and community-based outreach can promote informed use of testing and address existing patient beliefs and perceptions.

## CRediT authorship contribution statement

**Pranali G. Patel:** Writing – original draft, Formal analysis. **Marvin E. Langston:** Writing – review & editing, Writing – original draft, Formal analysis. **Justin X. Moore:** Writing – review & editing, Writing – original draft, Formal analysis. **Yunan Han:** Writing – review & editing, Writing – original draft, Formal analysis. **Marquita W. Lewis-Thames:** Writing – review & editing, Writing – original draft, Formal analysis. **Lindsay Fuzzell:** Writing – review & editing, Writing – original draft, Formal analysis. **Saira Khan:** Writing – review & editing, Writing – original draft, Formal analysis, Conceptualization.

## Consent to participate

Not applicable.

## Ethics approval and consent to participate

This study was exempt from institutional review board review due to the use of publicly available and de-identified data.

## Funding

SK was supported by 10.13039/100014571Siteman Cancer Center and the 10.13039/100007338Foundation for Barnes-Jewish Hospital. JXM was supported by the 10.13039/100006545National Institute on Minority Health and Health Disparities (Grant K01MD015304).

## Declaration of competing interest

The authors declare that they have no known competing financial interests or personal relationships that could have appeared to influence the work reported in this paper.

## Data Availability

Data Link has been provided in the manuscript

## Appendix A Supplementary data

Supplemental Tables 1-4 describe the cohort characteristics stratified by cancer history, gender, urban-rural status, physical activity, and obesity. Image 1
