# Supplementary material for: Beliefs and perceptions of genetic testing by clinical, demographic, and health characteristics: A U.S.-based study utilizing the health information national trends (HINTS) survey
Source: Prev Med Rep. 2025 Aug 8;57:103204. doi: 10.1016/j.pmedr.2025.103204 (PMC12359184; doi:10.1016/j.pmedr.2025.103204)
Supplement: Supplementary file 1 — Supplemental Tables 1-4 describe the cohort characteristics stratified by cancer history, gender, urban-rural status, physical activity, and obesity. [file mmc1.pdf]

Supplemental Table 1. Demographic Characteristics of U.S. Adults by History of Cancer—Health Information National Trends Survey 5, Cycle 4 (February 2020 -January 2020)

**Outcomes**	**Cancer survivors**	**No history of cancer**	
	**N (%)***	**N (%)***	***P*†**
**Gender**			
Men	237 (43.3)	1233 (49.7)	0.0833
Women	331 (56.7)	1701 (50.7)	
Missing	58	234	
**Age**			
18-34	12 (3.1)	472 (28.6)	<0.0001
35-49	45 (16.0)	656 (26.4)	
50-64	168 (32.2)	973 (27.3)	
65-74	208 (24.8)	660 (10.7)	
75+	185 (23.9)	352 (7.1)	
Missing	8	55	
**Rural Urban Status**			
Urban	543 (81.2)	2834 (88.6)	0.0086
Rural	83 (18.8)	334 (11.4)	
**Highest level of school completed**			
College Graduate	262 (28.6)	1391 (30.7)	0.4894
Not College Graduate	342 (71.4)	1688 (69.3)	
Missing	22	89	
**White**			
Yes	495 (88.3)	2188 (77.7)	<0.0001
No	105 (11.7)	749 (22.3)	
Missing	26	231	

**Ethnicity**			
Non-Hispanic	500 (92.9)	2370 (82.1)	<0.0001
Hispanic	53 (7.1)	534 (17.9)	
Missing	73	264	
**Combined annual household income**[#]			
Less than or equal to 75,000	364 (66.4)	1737 (56.5)	0.0062
More than 75,000	184 (33.6)	1129 (43.5)	
Missing	78	302	
**In a typical week, how many days do you do any physical activity or exercise of at least moderate intensity?**			
More than three days a week	287 (50.6)	1419 (44.5)	0.1280
Less than three days a week	326 (49.4)	1713 (55.5)	
Missing	13	36	
**Obesity status**			
Individuals living with obesity	199 (33.7)	1025 (33.2)	0.8950
Non-obese individuals	412 (66.3)	2057 (66.8)	
Missing	15	86	

**Presented as row weighted percentage.*
[#]Combined annual household income is in U.S. dollars
†P-values determined using either weighted χ^2
Frequency calculated among respondents using non-missing data.

Supplemental Table 2. Demographic Characteristics of U.S. Adults by Gender—
Health Information National Trends Survey 5, Cycle 4 (February 2020 -January 2020)

**Outcomes**	**Men**	**Women**	
	**N (%)***	**N (%)***	***P*†**
**Age**			
18-34	184 (29.1)	284 (24.8)	0.0003
35-49	252 (24.4)	415 (26.3)	
50-64	461 (27.9)	599 (27.4)	
65-74	371 (11.7)	432 (12.2)	
75+	194 (6.9)	280 (9.3)	
Missing	25	42	
**Rural Urban Status**			
Urban	1338 (89.1)	1803 (85.8)	0.0575
Rural	149 (10.9)	249 (14.2)	
**Highest level of school completed**			
College Graduate	672 (29.6)	914 (31.5)	0.0045
Not College Graduate	809 (70.4)	1122 (68.5)	
Missing	6	16	
**White**			
Yes	1105 (79.8)	1476 (77.5)	0.2161
No	320 (20.2)	489 (22.5)	
Missing	62	87	
**Ethnicity**			
Non-Hispanic	1168 (82.7)	1589 (84.4)	0.4037
Hispanic	238 (17.3)	327 (15.6)	
Missing	81	136	

**Combined annual household income**[#]			
Less than or equal to 75,000	809 (54.5)	1208 (59.3)	0.1022
More than 75,000	611 (45.5)	681 (40.7)	
Missing	67	163	
**In a typical week, how many days do you do any physical activity or exercise of at least moderate intensity?**			
More than three days a week	855 (57.3)	1056 (53.2)	0.1551
Less than three days a week	621 (42.7)	972 (46.8)	
Missing	11	24	
**Ever Had Cancer**			
Yes	237 (8.0)	331 (10.1)	0.0833
No	1233 (92.0)	1701 (89.9)	
Missing	17	20	
**Body Mass Index**			
Individuals living with obesity	466 (33.5)	691 (33.4)	0.9579
Non-obese individuals	1006 (66.5)	1301 (66.6)	
Missing	15	60	

Presented as row weighted percentage.
[#]*Combined annual household income is in U.S. dollars*
†*P-values determined using either weighted χ^2 or t tests.*
Frequency calculated among respondents using non-missing data.

Supplemental Table 3. Demographic Characteristics of the U.S. Adults by Rural-Urban Status—Health Information National Trends Survey 5, Cycle 4 (February 2020 -January 2020)

**Outcomes**	**Urban**	**Rural**	
	**N (%)***	**N (%)***	***P*†**
**Gender**			
Men	1338 (50.0)	149 (42.4)	0.0575
Women	1803 (50.0)	249 (57.6)	
Missing	294	32	
**Age**			
18-34	449 (27.5)	35 (16.7)	0.0076
35-49	639 (25.6)	64 (24.6)	
50-64	1009 (27.0)	133 (32.8)	
65-74	766 (11.8)	103 (13.0)	
75+	465 (8.0)	75 (13.0)	
Missing	107	20	
**Highest level of school completed**			
College Graduate	1525 (31.6)	138 (20.6)	0.0002
Not College Graduate	1783 (68.4)	276 (79.4)	
Missing	127	16	
**White**			
Yes	2349 (76.8)	358 (91.4)	<0.0001
No	814 (23.2)	53 (8.6)	
Missing	325 (7.3)<br>272	50 (7.7)<br>19	
**Ethnicity**			
Non-Hispanic	2540 (81.2)	354 (96.1)	<0.0001
Hispanic	570	26	
Missing	325 (7.3)	50 (7.7)	

**Combined annual household income**[#]			
Less than or equal to 75,000	1852 (56.4)	275 (65.9)	0.0890
More than 75,000	1218 (43.6)	103 (34.1)	
Missing	365	52	
**In a typical week, how many days do you do any physical activity or exercise of at least moderate intensity?**			
More than three days a week	1859 (55.8)	210 (50.3)	0.1836
Less than three days a week	1517 (44.2)	212 (49.7)	
Missing	59	8	
**Ever Had Cancer**			
Yes	543 (8.5)	83 (14.3)	0.0086
No	2834 (91.5)	334 (85.7)	
Missing	58	13	
**Obesity status**			
Individuals living with obesity	1086 (32.1)	151 (39.9)	0.0696
Non-obese individuals	2246 (67.9)	262 (60.1)	
Missing	103	17	

Presented as row weighted percentage.

[#]*Combined annual household income is in U.S. dollars*

†*P-values determined using either weighted χ^2 or t tests.*

Frequency calculated among respondents using non-missing data.

Supplemental Table 4. Demographic Characteristics of U.S. Adults by Physical activity and Obesity status—Health Information National Trends Survey 5, Cycle 4 (February 2020 -January 2020; U.S.)

**Outcomes**	**Physical Activity <3 days**	**Physical Activity ≥3 days**		**Individuals living with obesity**	**Individuals without obesity**	
	**N (%)***	**N (%)***	***P*†**	**N (%)***	**N (%)***	***P*†**
**Gender**						
Men	621 (47.0)	855 (51.1)	0.1551	466 (49.4)	1006 (49.2)	0.9579
Women	972 (53.0)	1056 (48.9)		691 (50.6)	1301 (50.8)	
Missing	136	158		80 (4.6)	201 (6.3)	
**Age**						
18-34	192 (25.9)	291 (26.7)	0.0001	132 (20.7)	346 (29.3)	0.0005
35-49	303 (24.3)	391 (26.4)		254 (30.6)	432 (23.1)	
50-64	508 (26.8)	625 (28.6)		416 (31.0)	703 (25.9)	
65-74	371 (11.3)	487 (12.5)		268 (10.7)	578 (12.3)	
75+	303 (11.8)	221 (5.8)		141 (6.9)	381 (9.4)	
Missing	52	54		26 (1.4)	68 (2.7)	
**Rural Urban Status**						
Urban	1517 (86.5)	1859 (88.9)	0.1836	1086 (85.3)	2246 (89.1)	0.0696
Rural	212 (13.5)	210 (11.1)		151 (14.7)	262 (10.9)	
**Highest level of school completed**						
College Graduate	619 (24.8)	1034 (35.1)	<0.0001	459 (23.9)	1171 (33.9)	0.0001
Not College Graduate	1052 (75.2)	974 (64.9)		745 (76.1)	1258 (66.1)	
Missing	58	61		33 (1.2)	79 (2.8)	
**White**						
Yes	1165 (75.8)	1514 (81.4)	0.0089	856 (78.6)	1802 (79.2)	0.8382
No	439 (24.2)	412 (18.6)		290 (21.4)	548 (20.8)	
Missing	125	143		91 (6.1)	158 (6.8)	

**Ethnicity**						
Non-Hispanic	1279 (81.8)	1585 (84.0)	0.4983	926 (81.7)	1910 (83.8)	0.4204
Hispanic	278 (18.2)	312 (16.0)		208 (18.3)	374 (16.2)	
Missing	172	172		103	224	
**Combined annual household income**[#]						
Less than or equal to 75,000	1062 (64.6)	1038 (51.5)	0.0001	747 (62.5)	1332 (54.6)	0.0291
More than 75,000	491 (35.4)	822 (48.5)		382 (37.5)	928 (45.4)	
Missing	176	209		108	248	
**In a typical week, how many days do you do any physical activity or exercise of at least moderate intensity?**						
More than three days a week	-	-	-	544 (44.2)	1483 (60.8)	<0.0001
Less than three days a week	-	-		677 (55.8)	996 (39.2)	
Missing	-	-		16	29	
**Obesity status**						
Individuals living with obesity	677 (41.2)	544 (26.3)	<0.0001	-	-	-
Non-obese individuals	996 (58.8)	1483 (73.7)		-	-	
Missing	56	42		-	-	
**Ever Had Cancer**						
Yes	287 (10.2)	326 (8.2)	0.1280	199 (9.3)	412 (9.1)	0.8950
No	1419 (89.8)	1713 (91.8)		1025 (90.7)	2057 (90.9)	
Missing	23	30		13	39	

Presented as row weighted percentage.
[#]*Combined annual household income is in U.S. dollars*
†*P-values determined using either weighted* χ^2 *or t tests.*
Frequency calculated among respondents using non-missing data.
